# Expression of a Barhl1a reporter in subsets of retinal ganglion cells and commissural neurons of the developing zebrafish brain

**DOI:** 10.1038/s41598-020-65435-w

**Published:** 2020-06-01

**Authors:** Shahad Albadri, Olivier Armant, Tairi Aljand-Geschwill, Filippo Del Bene, Matthias Carl, Uwe Strähle, Lucia Poggi

**Affiliations:** 1 Centre for Organismal Studies, University of Heidelberg, Heidelberg, Germany; 2 Institute of Biological and Chemical Systems, Biological Information Processing Karlsruhe Institute of Technology, Eggenstein-Leopoldshafen, Germany; 3 Institut de la Vision, Sorbonne Université, INSERM, CNRS, Paris, France; 4 Department of Cellular, Computational and Integrative Biology - CIBIO, University of Trento, Trento, Italy

**Keywords:** Developmental neurogenesis, Cell death in the nervous system, Neural stem cells, Differentiation, Reprogramming

## Abstract

Promoting the regeneration or survival of retinal ganglion cells (RGCs) is one focus of regenerative medicine. Homeobox Barhl transcription factors might be instrumental in these processes. In mammals, only *barhl2* is expressed in the retina and is required for both subtype identity acquisition of amacrine cells and for the survival of RGCs downstream of Atoh7, a transcription factor necessary for RGC genesis. The underlying mechanisms of this dual role of Barhl2 in mammals have remained elusive. Whole genome duplication in the teleost lineage generated the *barhl1a* and *barhl2* paralogues. In the Zebrafish retina, Barhl2 functions as a determinant of subsets of amacrine cells lineally related to RGCs independently of Atoh7. In contrast, *barhl1a* expression depends on Atoh7 but its expression dynamics and function have not been studied. Here we describe for the first time a Barhl1a reporter line *in vivo* showing that *barhl1a* turns on exclusively in subsets of RGCs and their post-mitotic precursors. We also show transient expression of *barhl1a:GFP* in diencephalic neurons extending their axonal projections as part of the post-optic commissure, at the time of optic chiasm formation. This work sets the ground for future studies on RGC subtype identity, axonal projections and genetic specification of Barhl1a-positive RGCs and commissural neurons.

## Introduction

BarH-like homeodomain (BARHL) transcription factors play crucial roles in the control of neural cell fate specification, migration, subtype identity acquisition and survival during development of the retina and the brain^1–7^. Studies have also implicated Barhl in neurodegenerative and neoplastic disorders^8,9^. Previous *barhl* studies have suggested that whole genome duplication (WGD) during vertebrate evolution generated two homologs of *barhl*: *barhl1* and *barhl2*^10^. In addition, studies in *Xenopus* and mouse suggest that *barhl2* (previously named *MBH1* and *XBH1*) is the only family member expressed in the retina of tetrapods^11,12^. Specifically, *barhl2* expression is found in both amacrine cells and retinal ganglion cells (RGCs) of the developing and mature retina^13,14^. Furthermore, Barhl2 is both sufficient and essential for determining the subtype specific identity of amacrine cells as well as to promote the maturation and survival of RGCs. Lastly, the expression of *barhl2* in RGCs appears to depend on Atoh7 (also known as Ath5) – a bHLH transcription factor required for the specification of RGCs in vertebrates^13–19^.

In Zebrafish, three *barhl* paralogs were found^4,7,10^. Studies based on both Barhl protein sequence analysis and conserved gene synteny between *barhl* genes suggest that they likely arise from the additional round of WGD in teleosts followed by the loss of one *barhl* paralog^10^. These studies also have revealed that *barhl1b* (previously called *barhl1* or *barhl1.1*) and *barhl1a* (previously called *barhl1.2*) are more closely related to the mammalian *barhl1*.  Conversely, the *barhl2* paralogue is more closely related to the mammalian *barhl2*^10^. Concordantly, similarly to its *barhl1* counterpart in tetrapods, *barhl1b* lost its retinal expression, maybe due to a redundant function with *barhl1a* and relaxed evolutionary pressure in its locus^4,10^. On the contrary, retinal expression of *barhl1a* and *barhl2* is retained, but these two paralogs appear to have diversified their function in retinal lineages^10^. In favour of this hypothesis, studies have shown that, similarly to the mammalian counterpart, Barhl2 is an amacrine cell subtype identity-biasing factor downstream of the transcription factor Ptf1a^20^. Furthermore, taking advantage of the zebrafish transgenesis combined with accessibility to 3D time-lapse imaging^21–25^ we have previously shown that *barhl2*-expressing amacrine subtypes consistently arise within the lineage of Atoh7 upon reproducible asymmetrical divisions of RGC progenitors^20^ (see also Fig. 9). Interestingly, while *barhl2* turns on exclusively in amacrine cells under the control of Ptf1a^20^, the expression of *barhl1a* depends on Atoh7 and appears therefore to be restricted to the ganglion cell layer (GCL)^10^. The generation pattern of individual *barhl1a*-expressing cells within the Atoh7 cell lineage, the lineage relationship among *barhl1a* and *barhl2* -expressing cells *in vivo* as well as the role played by Barhl1a in RGC genesis have so far remained unknown.

To address these questions, direct and dynamic visualization of *barhl1a*-expressing cells is needed. We used Tol2 transposase-mediated BAC transgenesis to generate a Barhl1a reporter line where *GFP* expression is driven by *barhl1a* regulatory regions. This reporter recapitulates *barhl1a* mRNA expression and helped us determine that *barhl1a* turns on exclusively in a sub-population of RGCs after the cell cycle exit of *atoh7-*expressing progenitors. In addition, the reporter allows us to visualize axonal connectivity derived from *barhl1a-*expressing neurons. Furthermore, we provide the first description of the expression dynamic of *barhl1a* in commissural neurons of the forebrain. This study thereby provides the foundation for further investigations of the role of Barhl1a transcription factors in nerve cell subtype identity acquisition and maintenance in this *in vivo* model system.

## Results

### *Barhl1a* in the retina is up-regulated in a subset of *atoh7*-expressing post-mitotic RGC precursors

**Figure 1:**
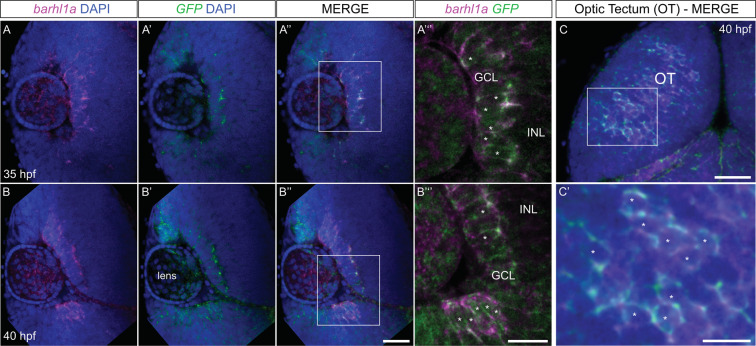
Expression of endogenous *barhl1a* is reflected by *barhl1a:GFP* in the retina and brain. (**A–B”’**) Confocal optical section in frontal view (dorsal is to the top) through the retina of a 35 hpf (A - A’’’) and 40 hpf (B - B’’’) embryo after FISH against endogenous *barhl1a* (A, B in magenta) and the *GFP* transgene (A’, B’ in green). Retinae are counterstained with DAPI (blue). (A”, B”) merging of the two channels shows co-localization of the two transcripts in the GCL. (A”’, B”’) magnifications of (A”) and (B’’) (white squares), respectively, without the DAPI channel. (**C**-**C’**) Confocal imaging into the tectum of a 40 hpf embryo after FISH and DAPI conterstaining shows co-localization of *barhl1a* with *GFP*. Images represent a Z-stack in dorsal view (anterior is to the top) into the optic tectum (OT). (C’) magnification of (C) (white square). Asterisks highlight regions with colocalizing signals. GCL: ganglion cell layer, INL: inner nuclear layer, OT: optic tectum. Scale bars: (A-A”, B-B”, C) 50 µm, (A’’’, B’’’, C’) 20 µm.

In order to visualize *barhl1a*-expressing cells *in vivo* we generated a *Tg(barhl1a:GFP)* transgenic line expressing the reporter eGFP under *barhl1a* regulatory genomic elements. To this end, a BAC spanning the *barhl1a* genomic locus was used to perform Tol2 transposon-mediated BAC transgenesis replacing *eGFP* with the *barhl1a* coding sequence (see Methods). The newly generated *Tg(barhl1a:GFP)* transgenic embryos displayed the distribution pattern of GFP as expected from *barhl1a* expression in the posterior thalamus and zona limitans intratalamica (ZLI)^4^ (Supplementary Fig. 1A). To further assess the reliability of the *Tg(barhl1a:GFP)* line, we compared the spatial expression pattern of *barhl1a* mRNA and *GFP* mRNA in *Tg(barhl1a:GFP)* embryos by double fluorescent *in situ* hybridization (FISH). Transcripts of both genes can be consistently found as shown, for example, at 35 and 40 hours post-fertilization (hpf), in the developing GCL as well as in the optic tectum (Fig. 1).

**Figure 2:**
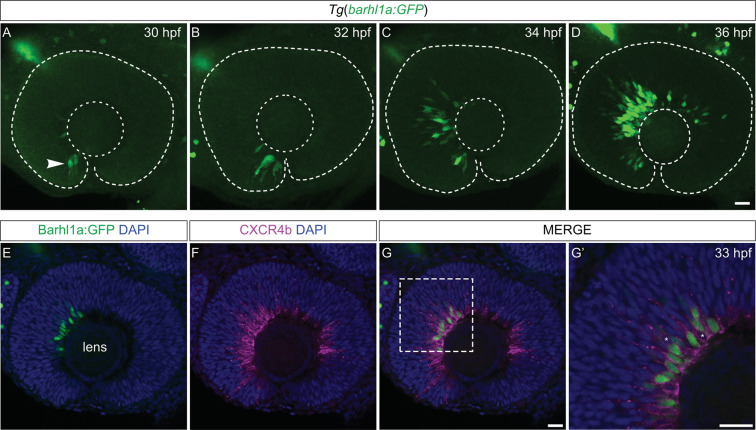
*Tg*(*barhl1a:GFP*) labels new-born RGCs. (**A**–**D**) Spatial-temporal distribution of *Tg*(*barhl1a:GFP*) expression in three different retinal developmental stages at 30 (**A**), 32 (**B**), 34 (**C**) and 36 (**D**) hpf. The GFP signal (green) becomes first evident at ~30 hpf in the ventral retina (white arrow in A) and subsequently follows the spatio-temporal wave of RGC differentiation. All images represent 1 µm Z-stacks in lateral view. (**E**–**G’**) Single 1 µm optical slice (Z-stack) of a 33 hpf *Tg*(*barhl1a:GFP*) embryo immunolabeled with anti-Cxcr4b (magenta) and anti-GFP (green) antibodies. (**E**) GFP is detected in cells located in the basal half of the neuroepithelium around the lens at 33 hpf. These are also labelled by Cxcr4b (magenta in F, G and zoomed insert G’), which mark post-mitotic RGCs and their precursors at the cell membrane. Asterisks in G’ highlight Cxcr4b-positive (GFP-negative) RGCs. All images represent lateral view, anterior is left, dorsal is top. Scale bars: 20 µm.

To directly investigate the dynamics of the appearance of individual Barhl1a:GFP-positive cells in the retina, we used 3D time-lapse imaging on *Tg(barhl1a:GFP)* embryos. Imaging of the developing retina of *Tg(barhl1a:GFP)* embryos for at least 20 hours revealed that GFP positive cells become first visible in the ventral retina at ~ 29-30 hpf and spread subsequently to the nasal, dorsal and temporal retina in a temporal and spatial fashion predictive of the wave of RGC differentiation (Fig. 2A–D and Supplementary Movie 1)^26^. Individual cells turning on *barhl1a:GFP* can be seen located either in the basal half or in the apical half of the neuroepithelium, from where they subsequently migrate basally until they reach the GCL (Fig. 2A–D and Supplementary Movie 1). We never observed Barhl1a:GFP cells migrating to the apical surface to undergo division. This suggests that *barhl1a:GFP* turns on in RGC precursors after the terminal division of *atoh7*-expressing neuroepithelial cells. Immuno-labelling of *Tg(barhl1a:GFP)* embryos with an antibody to Cxcr4b, a marker of post-mitotic RGCs^27^ further supports our notion that *barhl1a:GFP* is expressed in RGCs and their post-mitotic precursors (Fig. 2E–G’).

**Figure 3:**
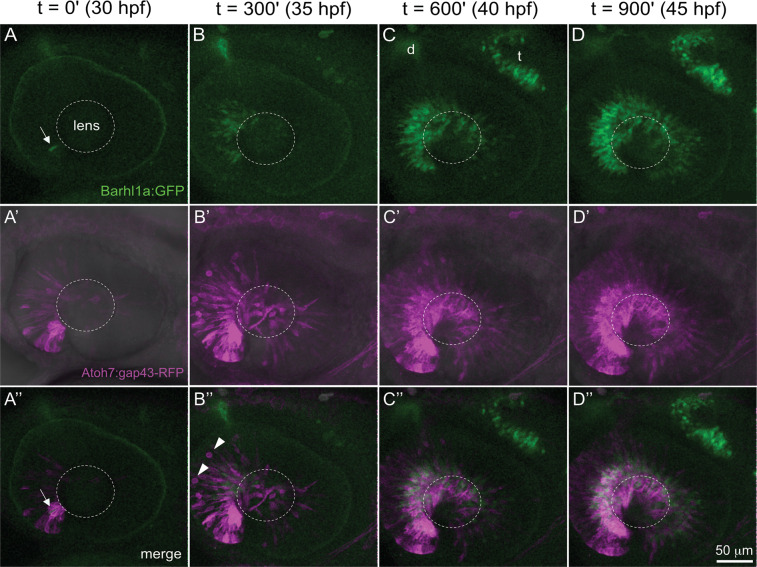
Time course of *barhl1a:GFP;atoh7:gap43-RFP* expression. Frames from a time-lapse movie in the retina of a *Tg*(*barhl1a:GFP;atoh7:gap43-RFP*) double transgenic embryo imaged from ~30 hpf to ~45 hpf. Each time-frame represents a projection of Z-stacks in lateral view. Anterior is to the left, dorsal is to the top. Time-points (t) are t = 0 minute (’) at 30 hpf (**A**-**A”**), t = 300’~35 hpf (**B**-**B”**), t = 600’~40 hpf (**C**-**C”**) and t = 900’~45 hpf (**D**-**D”**). The first GFP-positive cells (in green) can be detected at ~ 30 hpf (t = 0) in the anterior-ventral retina (white arrow in A, A’’), when the Atoh7:gap43-RFP signal (in magenta) has already spread across the nasal retina at this developmental stage. The wave of Barhl1a:GFP follows the wave of Atoh7:gap43-RFP across the dorsal and temporal retina (**B–D”**), always remaining confined to the basal half of the retinal neuroepithelium. White arrowheads in (B”) point at RFP-positive (GFP-negative) cells rounding up at the apical surface before mitotic division. Barhl1a:GFP expression can also be observed in the developing optic tectum (t, C) and diencephalon (d, C). The dotted circles highlight the position of the lens. Scale bar: 50 µm.

**Figure 4:**
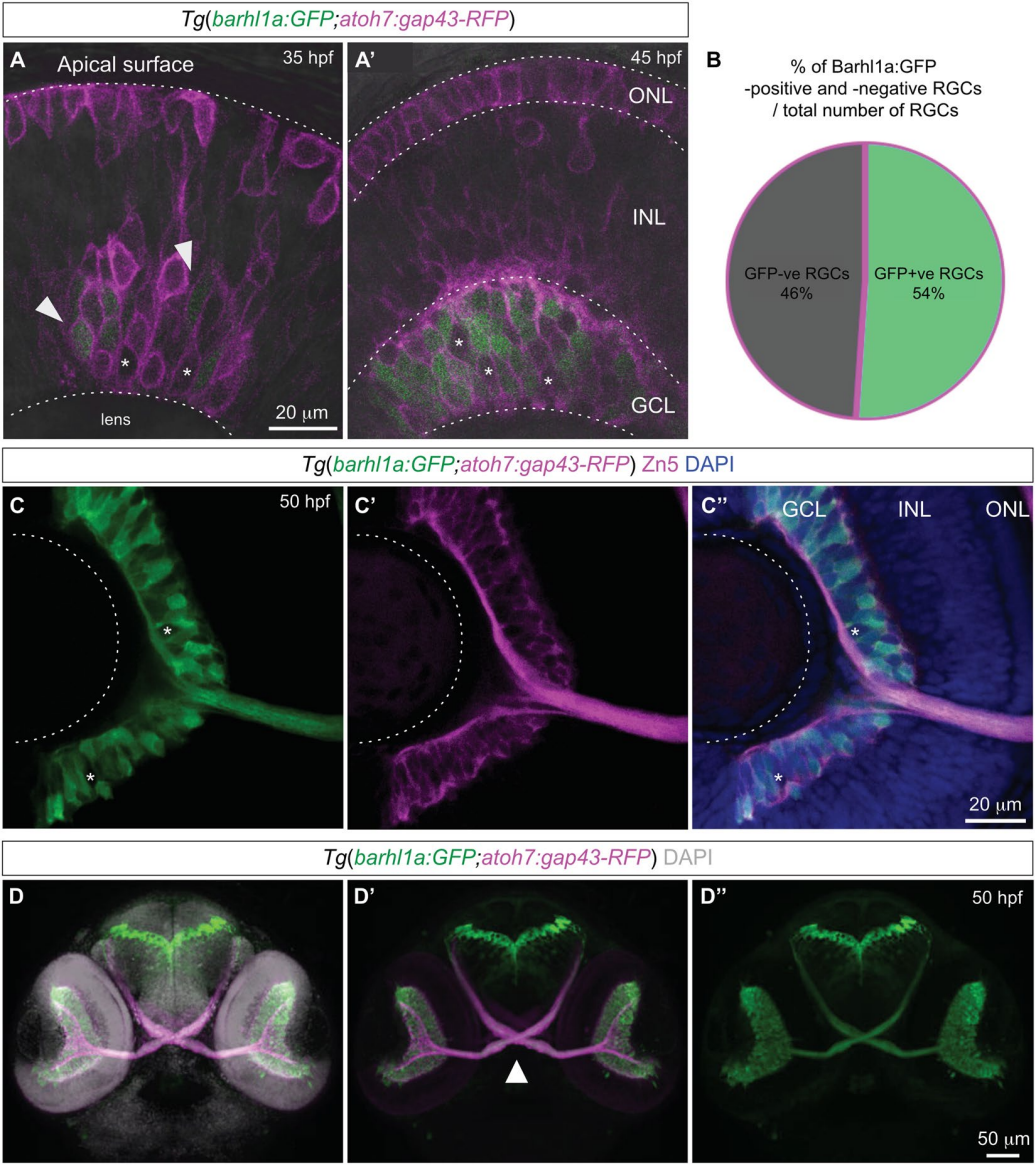
Barhl1a:GFP labels a population of new-born RGCs and their axons. (**A**,** A’**) Single optical slices (Z-stacks) extracted from a movie frame of a retina of a Tg(*barhl1a:GFP;atoh7:gap43-RFP*) embryo at ~35 and ~45 hpf, respectively, showing the distribution of Barhl1a:GFP (green) and Atoh7:gap43-RFP (magenta) cells across the basal-apical surface of the retinal neuroepithelium. For both images anterior is to the left, dorsal is to the top. The outline of the retina is shown in the gray channel. Asterisks highlight the GFP-negative (gap43-RFP -positive) cells whereas the arrowheads point at the GFP positive cells. (**B**) Quantification of the percentage of GFP-positive (GFP + ve) and GFP-negative (GFP-ve) cells performed at 50 hpf revealed that 54% of RGCs are expressing Barhl1a:GFP (1391 total counted cells from n = 7 retinae). (**C**-**C”**) A single optical slice (Z-stack) in frontal view (dorsal is top) through the retina of a 50 hpf, *Tg(barhl1a:GFP;atoh7:gap43-RFP)* embryo counterstained with DAPI (C”), anti-GFP (C, green) and the RGC marker anti-Zn5 (C’, magenta) to brightly label both Barhl1a:GFP and the GCL. Asterisks highlight Barhl1a:GFP-negative (gap43-RFP/Zn5 -positive) RGCs. (**D**-**D”**) Frontal view of a 3D reconstruction of the head of an *Tg(barhl1a:GFP;atoh7:gap43-RFP)* embryo at 50 hpf (dorsal is to the top). Atoh7:gap43-RFP labelled retina and optic nerve are shown in magenta. At this stage, Barhl1a-GFP cells (green) have fully differentiated and extended their axons out of the retina forming the optic nerve. The optic nerves cross contra-laterally to form the optic chiasm (white arrowhead, D’) and reach their targets in the brain. The embryo has been counterstained with DAPI as shown in gray in (D). ONL, outer nuclear layer; INL, inner nuclear layer; GCL, ganglion cell layer. Scale bars: (A-A’) 20 µm, (C-C”) 20 µm, (D-D”) 50 µm.

To further investigate expression of *Tg(barhl1a:GFP)* with respect to *atoh7* we generated a *Tg(barhl1a:GFP;atoh7:gap43-RFP)* double transgenic line in which the Atoh7:gap43-RFP labels all *atoh7*-expressing progenitors and their derived retinal cell types, including *barhl2-*expressing amacrine cells^20,28^. Studies have shown that Atoh7:gap43-RFP becomes first visible in the anterior ventral retina already at 27–28 hpf, underlying the onset of retinal neurogenesis^20,28,29^. Consistently, *GFP* expression from the Barhl1a reporter could be seen appearing at ~30 hpf, hence with two or three hours delay after the onset of *atoh7:gap43-RFP* expression (Fig. 3A–A”). Likewise, consistently with the idea of Barhl1a:GFP being restricted to post-mitotic RGCs, *GFP* expression was always seen in the basal half of the neuroepithelium whilst it remains excluded from the retinal apical surface where mitosis takes place (Fig. 3B–D” and 4A-A’). At 50 hpf, the GFP signal remains strong in the RGCs and their axons that have extended along the optic tract (Fig. 4C,D” and Supplementary Movie 2).

Imaging in the retina of *Tg(barhl1a:GFP;atoh7:gap43-RFP)* embryos also revealed that not all Atoh7:gap43-RFP cells in the GCL appear as Barhl1a:GFP positive, as it would be expected if Barhl1a labels a subpopulation of RGCs (see asterisks in Figs. 2G’, 4A–A’,C–C”). To assess this, Barhl1a:GFP positive cells were counted in the central retina of 50 hpf, *Tg(barhl1a:GFP;atoh7:gap43-RFP)* embryos immunostained against GFP and Zn5 (also known as Alcama/DM-GRASP/Neurolin/Zn8^30^), a cell adhesion molecule that is transiently found on the entire surface of differentiated RGCs, and is therefore a suitable marker for labeling all RGCs^30^. Quantification of the Barhl1a:GFP nuclei shows that, on average, only about 54% of the RGC population (1391 cells, n = 7 retinae) is also Barhl1a:GFP positive (Fig. 4B and Supplementary Table 1).

**Figure 5:**
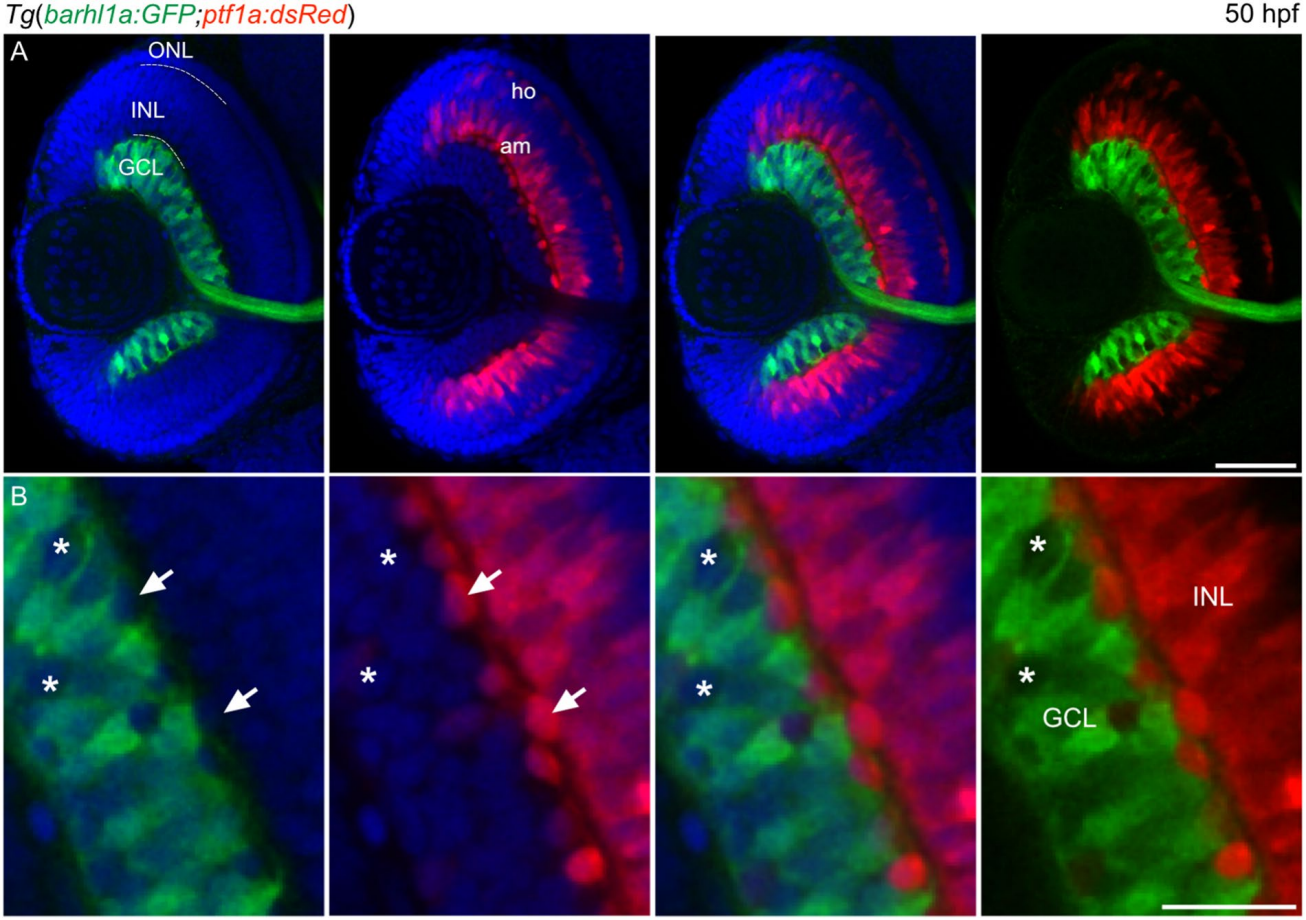
Barhl1a and Ptf1a reporter expressions are mutually exclusive. (**A–B**) Confocal imaging through the retina of a 50 hpf *Tg*(*barhl1a:GFP;ptf1a:dsRed*) embryo fixed and counterstained with DAPI (blue). Each image represents a single Z-stack in frontal view. Anterior is to the top. (**B**) always represents a higher magnification of (**A**). GFP-positive (green) cells are detected in the GCL whilst DsRed-positive cells (red) are detected in the amacrine (am) cells and horizontal (ho) cells of the inner nuclear layer (INL). White arrows point at DsRed-positive amacrine cells displaced in the GCL; which are always GFP-negative. Asterisks highlight the GFP-negative cells in the GCL; which are also DsRed-negative (**B**). GCL, ganglion cell layer; INL, inner nuclear layer; ONL, outer nuclear layer; am, amacrine cells; ho, horizontal cells. Scale bars: (**A**) 50 µm, (**B**) 20 µm.

Besides RGCs, the GCL also contains displaced amacrine cells. These displaced amacrine cells are however present in very low numbers, which would not largely affect the percentage of Barhl1a:GFP RGCs. We aimed nevertheless to rule out the possibility that some of the GFP-negative cells in the GCL may be displaced amacrine cells. To this end we generated a *Tg(barhl1a:GFP;ptf1a:dsRed)* double transgenic line, in which the *ptf1a:dsRed* transgene labels all amacrine and horizontal cells^31–33^ (Fig. 5). Analysis of the retinae of *Tg(barhl1a:GFP;ptf1a:dsRed)* embryos at 50 hpf revealed that expression of the two reporter genes are mutually exclusive, with all displaced amacrine cells expressing the Ptf1a reporter being Barhl1a:GFP-negative (Fig. 5). This further confirms that expression of *barhl1a* is restricted to a RGC population.

We conclude that *barhl1a* turns on in about half the RGC precursors. The other half of the *atoh7*-expressing RGC precursors would then differentiate without the direct contribution of Barhl1a in the retina.

### Expression of the Barhl1a reporter in the brain labels diencephalic commissural neurons and their mitotic precursors

**Figure 6:**
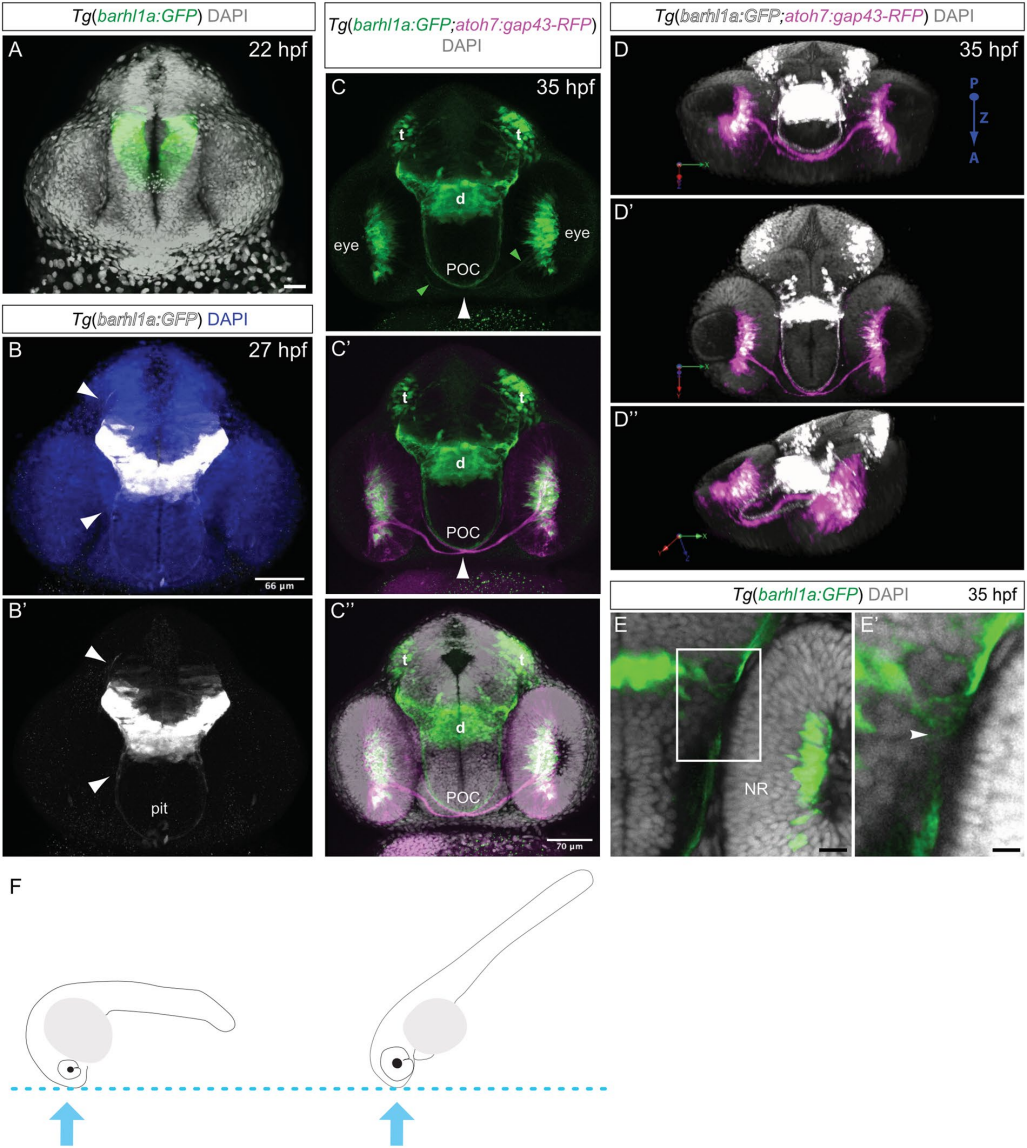
Developmental dynamic of Barhl1a:GFP cells in the diencephalon. Images represent projections of Z-stack (**A–C”, E, E’**) and 3D reconstructions (**D–D”**) of the anterior brain of fixed embryos at 22 (**A**), 27 (**B-B’**) and 35 hpf (**C-C”, D-D”** and **E-E**’) counterstained with DAPI. Images are in frontal view, dorsal is to the top. (**A**) Frontal view of the anterior brain of a *Tg(barhl1a:GFP)* transgenic embryo at 22 hpf showing Barhl1a:GFP (in green) in the presumptive diencephalon. DAPI staining is shown in gray. (**B**-**B’**) Frontal view of a *Tg(barhl1a:GFP)* transgenic embryo at 27 hpf. DAPI in (B) is shown in blue. White arrowheads point at Barhl1a:GFP projections (in gray) that extend and cross the midline ventrally and dorsally, respectively. The fibers that cross ventrally form a bundle of fibers along the presumptive optic tract. At this stage, few GFP-positive cells likely corresponding to pituitary cells can also be detected (pit, B’). (**C**-**C”**) Frontal view of an 35 hpf *Tg*(*barhl1a:GFP;atoh7:gap43-RFP*) double transgenic embryo. DAPI staining is shown in gray in C’. Barhl1a:GFP cells (in green) can be seen in tectal (t) and diencephalic (d) domains as well as in the eye. Bundles of Barhl1a:GFP fibers can be clearly seen crossing the ventral midline along the post optic commissure (POC). At this stage, the Atoh7:gap43-RFP positive RGCs (magenta) extend their axons out of the retina to form the optic nerve. The optic nerves cross contralaterally at the optic chiasm (white arrowhead) in close proximity to the diencephalic GFP-positive fibers forming the POC. The green arrowheads in (C) point at the few Barhl1a:GFP RGC fibers (in green), which are contained within the big bundle of magenta RGC fibers shown in C’ and C”. (**D**-**D”**) 3D reconstruction and turnaround of the anterior head region of a double transgenic *Tg*(*barhl1a:GFP;atoh7:gap43-RFP*) embryo at 35 hpf highlighting the optic nerves (in magenta) alongside the Barhl1a:GFP fibers (in gray). DAPI staining is in gray. In all three panels the view is from the posterior side of the stack/embryo; P, posterior; A, anterior; Z, z-axis. (**E**-**E’**) Enlarged confocal image (E) and its magnification (E’) of a frontal view of an *Tg*(*barhl1a:GFP*) transgenic embryo highlighting the fibers originating from Barhl1a:GFP cells located in the diencephalon (arrowheads). (**F**) Schematic cartoons showing approximate embryo orientation and plane of confocal imaging. Pit, pituitary. Scale bars: (A) 28 µm, (B-B’) 66 µm, (C-C”) 70 µm, (E) 20 µm, (E’) 50 µm.

Prior to Barhl1a reporter activity in the retina, Barhl1a:GFP-positive cells can be seen starting from ﻿10-14 somites in the forebrain, in locations corresponding to the previously reported expression of *barhl1* mRNA in diencephalic domains of mammals, *Xenopus* and fish^1,3,4,10,34^ (Fig. 6A,B-B’ and Supplementary Movie 3). These diencephalic domains have been shown to encompass the prospective preoptic area, thalamus and posterior tuberculum/posterior hypothalamus^1,3,4^. Subsequent GFP signals extend to more posterior domains, such as the ventral midbrain, pretectal/tectal area and the hindbrain^3,4,35^ (Fig. 6C-C” and Supplementary Movie 4). Finally, imaging of the forebrain of *Tg(barhl1a:GFP)* embryos additionally revealed previously not reported *barhl1a* expression in few cells likely corresponding to pituitary cells (Fig. 6B-B’ and Supplementary Movie S4).

The Barhl1a reporter line offers the advantage to visualize not only the development of neuron cell bodies in the retina and brain but also of their axonal processes *in vivo* (Fig. 6). Imaging analysis of the brain of *Tg(barhl1a:GFP)* embryos indeed revealed, besides the optic chiasm, other two early commissural tracts deriving from *barhl1a*-expressing cells. A first bundle of GFP positive fibers become visible at around 20-22 hpf, extending on both sides of the ventral-anterior diencephalon towards the midline, and forming a commissure around the presumptive optic tract (Fig. 6B-B’ and Supplementary Movie 5). The first RGCs become postmitotic in the retina only at around 27 hpf^26^; we can therefore exclude that these Barhl1a:GFP-labelled axons crossing the ventral midline are RGC axons. Time-lapse imaging analysis using a lateral view further highlighted the temporal and spatial dynamics of the development of this ventral commissure in *Tg(barhl1a:GFP)* embryos, which appear before the optic chiasm and is located immediately posterior and dorsal to it (Supplementary Movie 6 and see also Supplementary Movie 4). These observations are consistent with Barhl1a:GFP-positive ventral commissure likely contributing to the previously described post optic commissure (POC)^36–39^. We then generated a BAC-based *barhl1a:gal4* (*barhl1.2:gal4-vp16*) DNA construct and injected it in one-cell stage *Tg*(*UAS:RFP;cry:GFP*) embryos, to obtain mosaic expression of *RFP* (Supplementary Movie 7). Single Barhl1a:gal4;UAS:RFP cells were seen extending their fibers along this POC; which are likely located in the caudal diencephalon, where commissural neurons have been previously described (Supplementary Movie 7)^36–42^. Furthermore, we found that *barhl1a* expression in this region overlaps with the expression of *lmx1b1*, a marker of the presumptive caudal diencephalon/posterior tuberculum in which commissure neurons have been reported (Supplementary Fig. S1B-B”)^43,44^. Remarkably, once the POC is fully formed and the optic nerves have reached their final destination, the Barhl1a:GFP commissural fibers are no longer visible (Fig. 4D-D” and Supplementary Movies 2). Therefore, the developmental spatial-temporal time of the appearance of the *barhl1a:GFP* expression in the POC tightly correlates with the formation of the optic tract.

**Figure 7:**
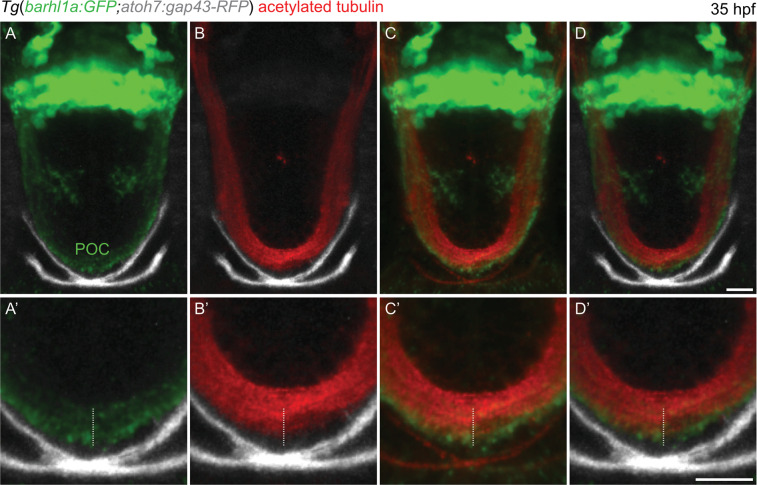
Barhl1a:GFP fibers co-localize with the neuronal projection marker acetylated Tubulin, a marker for the POC. (**A**–**D**) single optical sections (Z-stacks) through the anterior brain of a 35 hpf, *Tg*(*barhl1a:GFP;atoh7:gap43-RFP*) embryos immunostained with acetylated-Tubulin labelling the POC and its fibers (in red). Frontal view, dorsal is to the top. Barhl1a:GFP cells and their projections are shown in green whilst the optic nerves labelled by Atoh7:gap43-RFP are shown in gray. The optic nerves from each eye cross at the optic chiasm site, ventral and anterior to the POC, to then proceed along the optic tract. (**A’**–**D’**) higher magnification scan through the POC of the same embryo reveals partial overlap of the acetylated-Tubulin-labelled POC (highlighted by the white dashed line) and the Barhl1a:GFP fibers. Scale bars: (A–D) 50 µm, (A’–D’) 20 µm.

To further investigate the location of the Barhl1a:GFP-labeled diencephalic commissure with respect to the optic chiasm, we performed imaging in the brain of *Tg(barhl1a:GFP;atoh7:gap43-RFP)* embryos at 35 hpf (Fig. 6C–E’ and Supplementary Movie S4). At this developmental stage, Atoh7:gap43-RFP -positive RGC axons can be clearly identified; which exited the retina and crossed the ventral midline to form the optic chiasm^45^ (Fig. 6C’,D”). Conversely, only very few of these gap43-RFP -positive axons are also Barhl1a:GFP-positive (Fig. 6C-C”), further suggesting that the birth and/or maturation of Barhl1a:GFP RGCs is delayed with respect to the first wave of RGC genesis. Rotating the reconstructed 3D volume further highlighted the two contiguous but distinct commissures identified by the Atoh7 and Barhl1a reporters, respectively (Fig. 6D-D”). Lastly, *Tg(barhl1a:GFP;atoh7:gap43-RFP)* embryos were co-labeled immuno-histochemically with antibodies against acetylated Tubulin and GFP. The GFP signal overlaps, *albeit* only partially with that of acetylated Tubulin in the POC fibers (Fig. 7), further indicating that the Barhl1a:GFP cell projections contribute to this diencephalic commissure.

Notably, another bundle of Barhl1a:GFP positive axons could be seen at around 27 hpf, which extend dorsally and cross the midline at the presumptive rostral border of the developing tectum (Fig. 6B,C” and Supplementary Movie 6). Based on their temporal appearance and anatomical position, these commissural fibers are likely to grow along the path pioneered by the posterior commissure (PC) and might be therefore residing in the ventral midbrain or pretectal nuclei (Supplementary Movie 7)^36,38,42^.

**Figure 8:**
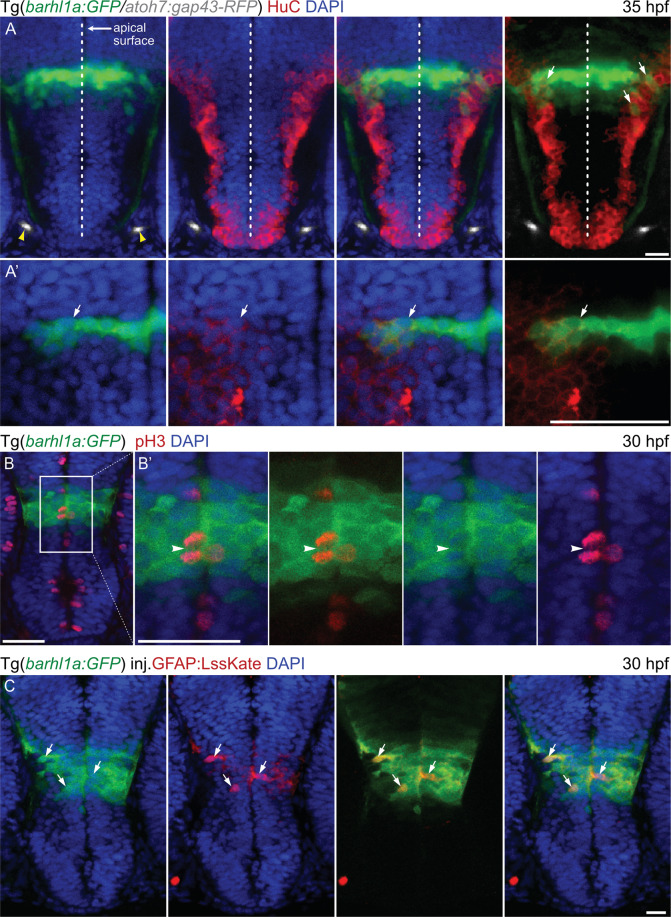
Barhl1a:GFP in the preoptic diencephalon marks populations of mitotic progenitors and differentiating neurons. Images represent single confocal Z-stacks through the diencephalon of *Tg*(*barhl1a:GFP;atoh7:gap43-RFP*) embryos at 35 hpf (**A, A’**) and 30 hpf (**B, C**). Frontal view, dorsal is up. Embryos in (**A**-**B’**) have been immunohistochemically stained with anti-GFP, anti-HuC (**A**,** A’**) and anti-pH3 (**B**,** B’**) antibodies (in red) as well as counterstained with DAPI (in blue). (A’) magnifications of the images in (A) in correspondence with the *barhl1a:GFP* expressing cell bodies. (A) yellow arrowheads point at the optic nerves from each side marked by Atoh7:gap43-RFP (in gray). White arrows point at Barhl1a:GFP cells co-labelled with HuC, a marker of differentiating neurons located in the basal half of the neuroepithelium. In (B) and its magnification (**B’**), Barhl1a:GFP cells are also co-labelled by pH3, which marks mitotic neurons located at the apical surface of the neuroepithelium (white arrowhead in B’). (**C**) 30 hpf *Tg*(*barhl1a:GFP*) embryo injected with the *gfap:lssKate* DNA construct shows sparse LssKate labelling in the diencephalon (in red); which co-localize with GFP (green). Scale bars: (A–C) 50 µm, (A’,B’) 20 µm.

In the pseudostratified neuroepithelium of the neural tube, post-mitotic neurons and glia are generated from progenitor cells ﻿that span the entire thickness of the neuroepithelium, from the basal to the apical surface. The nuclei of these progenitor cells undergo interkinetic nuclear migration, with mitotic nuclei being located at the apical surface whilst differentiating cells migrate to more basal locations^46,47^. Since Barhl1a:GFP-positive cells span the entire thickness of the diencephalic neuroepithelium, we asked whether these cells would be found at different developmental phases of neurogenesis. *Tg(barhl1a:GFP)* neurons were firstly labelled immune-histochemically with a HuC/Elav antibody labelling the cell body of differentiated neurons^48^. Overlap of HuC/Elav and GFP occurs in cells located further away from the apical (ventricular) surface, indicating that they are post-mitotic cells (Fig. 8A,A’). Conversely, GFP positive cells immediately adjacent to the apical ventricular surface expressed the mitotic marker phospho-histone 3 (pH3)^49^, indicating that they are mitotic precursors, as well as could be seen undergoing mitotic divisions (Fig. 8B-B’). Neuronal progenitors in the zebrafish neuroepithelium have been reported to express markers of radial glia, such as the glial acidic fibrillary protein (GFAP)^48,50^. Mosaic expression of an *gfap:lss-kate* construct, where the promoter of *gfap* has been cloned upstream of the *lss-mKate1* gene, highlighted GFAP:Lss-mKate1/Barhl1a:GFP double positive cells at different apical-basal distances within the thickness of the neuroepithelium (Fig. 8C).

We conclude that, unlike in the neural retina, where *barhl1a* expression is turned on post-mitotically in RGC precursors, expression of the Barhl1a reporter in the diencephalon marks populations of progenitor/precursor cells undergoing cell division and neuronal and glial differentiation.

In summary, the observed spatial-temporal dynamics of Barhl1a:GFP are consistent with Barhl1a being a subtype identity factor for populations of commissural neurons in the brain as well as in the retina. The Barhl1a reporter indeed specifically labels particular neuronal subsets in the forebrain and their extending axons, which form commissures across brain hemispheres. These *barhl1a:GFP*-expressing axons follow pre-existing embryonic commissures: the POC, the PC and the optic chiasm in a stereotypic pattern that is reminiscent of the previously described developmental sequence of the formation of archetypal tracts^36,37,51,52^.

## Discussion

*Barhl* genes have a crucial role in the control of neuronal subtype acquisition and maintenance during development and in the adult^1,2,5,6,8,15,53^. Loss of Barhl2 protein in the postnatal mammalian retina causes programmed cell death of more than 50% of RGCs^14^. Therefore, a better understanding of the Barhl function is essential to prevent RGC death and/or enable RGCs and axon regeneration. However, the mammalian Barhl2 is concomitantly expressed in amacrine cells and it is required for their subtype identity acquisition^14,15^. Untangling these two concurrent functions of Barhl2 might therefore be challenging in mammals. Barhl proteins play evolutionarily conserved roles in retinal cell type maturation^13,20^. Furthermore, the two zebrafish *barhl* paralogues genes, *barhl1a* and *barhl2*, exhibit complementary expression in the retina^10^, which together resemble mammalian Barhl2 expression.

We here started to explore whether it could be possible to disentangle the dual function of the single mammalian Barhl2 gene in the retina, by analyzing its zebrafish orthologous genes. With the generation of the *Tg(barhl1a:GFP)* transgenic line we established an essential resource. This line allowed us to provide for the first time a comprehensive description of the dynamic *barhl1a* expression *in vivo*. Our study confirms and extends previous findings by showing that *barhl1a:GFP* turns on exclusively in RGCs (most likely downstream of Atoh7^10^). Time-lapse imaging of individual Barhl1a:GFP-positive RGCs further enabled us to show that these cells are postmitotic neuro-epithelial cells, with their nuclei migrating from the apical half of the neuroepithelium towards the GCL.

Interestingly, these Barhl1a:GFP RGCs represent only about 54% of the RGCs population, which appear to display distinct retinotectal projections. There is a delay in the appearance of Barhl1a:GFP-positive axons compared to the appearance of Atoh7:gap43-RFP axons. One explanation for this delay might be that Barhl1a:GFP cells comprise a population of later-born RGCs, which grow their axons along the optic tract pioneered by the early-born Atoh7-positive/Barhl2-negative RGCs^52^. Alternatively, the delayed onset of GFP could also reflect the requirement of *barhl1a* expression for the late maturation and/or maintenance of RGCs. Conclusions cannot be drawn here and future studies using the *Tg(barhl1a:GFP)* line and *barhl1a*-inducible Gal4 constructs may be used to investigate the distinct molecular signature, morphology, dendritic patterns and retinotopic targets of Barhl1a RGCs.

**Figure 9:**
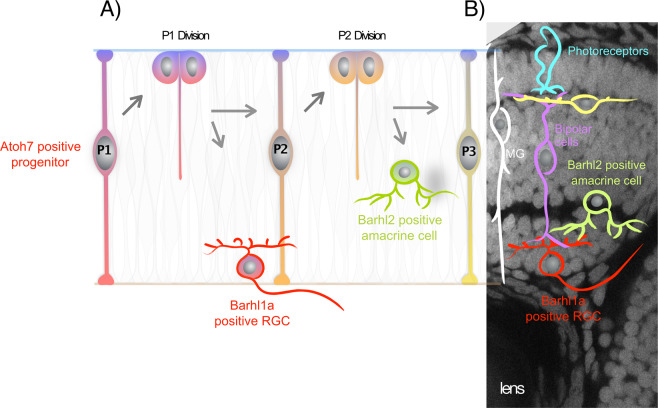
Hypothetical scenario describing Barhl1a RGCs and Barhl2 amacrine cells as clonally related retinal cells forming specific synapses. In this hypothetical model, (**A**) an asymmetric self-renewing division of an *atoh7*-expressing RGC progenitor gives rise to one Barhl1a-RGC and one Barhl2-amacrine cell sibling (see also^20^). (**B**) Cartoon representing the main retinal cell types organized into the three retinal nuclear layers; which are highlighted by the DAPI staining (in grey) of the retinal optical section from a 72 hpf embryo in the background. MG, Müller glial cells.

We have previously shown that about 58% of amacrine cells express *barhl2* in the Zebrafish retina, all of which seem to arise from the asymmetric divisions of *atoh7*-expressing progenitors. Moreover, Zebrafish Barhl2 is both necessary and sufficient for subtype identity acquisition of these amacrine cells downstream of Ptf1a^20^. Given the remarkably similar temporal and subtype-restricted expression pattern of *barhl1a*, it will be interesting in future studies to examine if and how Barhl1a determines particular RGC subtype identities and retinotopic projections, and whether these subtypes tend to have recurrent clonal relationships and connectivity patterns with their lineally-related Barhl2 amacrine subtypes (Fig. 9). Lastly, the fact that Barhl1a RGCs and Barhl2 amacrine cells are lineally related (that is: they arise from the same type of progenitor) suggests that these retinal cells share gene expression profiles and/or epigenetic ancestry. It will be exciting to explore whether such insights gained from a genome duplication in zebrafish will allow to narrow down the shared and distinct gene networks that are relevant for the role of Barhl in the maturation of RGCs and amacrines as well as in the survival of RGCs in the mammalian retina. Understanding this could be instrumental, for instance, for the identification of key factors whereby reprogramming of lineally-related retinal cell types, such as Barhl-dependent amacrines and RGCs, could be achieved to prevent RGC death or encourage their regeneration from amacrine cells^54^.

Unlike in the retina, where Barhl1a:GFP is restricted to post-mitotic neurons, *barhl1a:GFP* in the brain is turned on in mitotic progenitors and retained in their post-mitotic daughters. Using time-lapse analysis in the brain of *Tg(barhl1a:GFP)* embryos we discovered for the first time that Barhl1a:GFP in the diencephalon labels small populations of cells contributing to the POC and the PC tracts. We also found partial overlapping expressions of *barhl1a* and *lmx1b1*, a regulator of dopaminergic neurons^44^. It will be intriguing to assess in future studies whether these neurons co-expressing *lmx1b1* and *barhl1a* are the ones from which such commissures arise. Also interestingly is the finding that the onset of Barhl1a:GFP-labeled POC fibers coincides with the onset of RGC axon maturation and is transient, *i.e*. it disappears when the optic tract is fully differentiated. Lastly, it was also intriguing to find that, whilst the Barhl1a:GFP labelled POC neurons also express GFAP, a marker of progenitors and glial cells^48,50^, the Barhl1a:GFP labelled POC fibers only partially co-localize with the POC axonal marker acetylated tubulin. Future studies will assess the precise (glial and/or neuronal) identity of Barhl1a:GFP commissural fibers; they will also assess whether the remarkably harmonized developmental pattern between the Barhl1a:GFP-labeled POC and optic chiasm may underlie a molecular cross-talk required for the proper formation of the optic tract.

In conclusion, our novel experimental tools and insights into the spatial-temporal dynamics of Barhl1a:GFP in the retina and brain provide a fundamental framework for further investigation of Barhl1a-specific RGCs and their retinotectal RGC projections as well as of Barhl1a-commissural projection neurons *in vivo*. Such investigations are likely to be relevant to dissect BarHl functions in mammals and to understand and ameliorate the pathophysiology of BarHl-linked diseases.

## Methods

### Animals and ethic statement

Fish were maintained at 26–28 °C and embryos raised at 28.5 °C and staged as described previously^55,56^. Fish were housed in two facilities: Fish facility at COS, University of Heidelberg, Germany; fish facility at CIBIO, University of Trento, Italy. Each facility is under supervision of and in accordance with local animal welfare agencies and European Union animal welfare guidelines (Tierschutzgesetz 111, Abs. 1, Nr. 1; Regierungspräsidium Karlsruhe and the Italian Ministry of Health - permit no.: 151/2019-PR). As zebrafish sex cannot be determined until they have reached age of reproduction, embryos of either sex were used exclusively before free-feeding stages. Embryos used for whole-mount imaging were treated with 0.0045% 1-phenyl-2-thiourea (Sigma) to delay pigment formation. Lines used in this study were generated in the zebrafish wild type background (AB/AB or AB/WIK). The fish facility is under the supervision of the local representative of the Animal Welfare Agency.

### Fish lines and constructs

For the generation of the *Tg(barhl1a:GFP)* line, the BAC clone CH120-215H7 (CHORI, BACPAC, Oakland, CA, USA) was modified using the *flpe recombinase* system in EL250 cells^57^. The coding sequence of eGFP with a SV40 polyA site was inserted within the first exon of the *barhl1.2* genomic sequence and the tol2-AmpR cassette was inserted in the TARBAC2.1 backbone by PCR. The modified BAC was injected together with *tol2* mRNA into one cell-stage zebrafish embryos to generate a stable transgenic line.

Along with the newly generated and validated *Tg*(*barhl1a:GFP*) line, five other transgenic lines expressing *GFP*, *dsRed* and *gap43-RFP* under the control of different promoters were used in this study: the previously published *Tg*(*atoh7:gap43-RFP*)^28^; the derived outcrossed line *Tg*(*barhl1a*:*GFP;atoh7:gap43-RFP*); *Tg*(*barhl1a:GFP;ptf1a:dsRed*) generated by outcrossing *Tg*(*barhl1a:GFP*) and *Tg*(*ptf1a:dsRed*) line *Tg((-5.5ptf1a:DsRed)ia6)* previously published^20^ and the previously published *Tg*(*UAS:RFP;cry:GFP*) transgenic line^58^. Embryos carrying both transgenes were screened for expression of the red and green reporters using an Olympus MVX10 macrofluorescence binocular.

The *barhl1a:gal4* bacterial artificial chromosome (BAC) construct was generated as follows: a BAC spanning the *barhl1a* genomic locus (CH211-215C18, BACPAC Resources Center) was used to perform Tol2 transposon-mediated BAC transgenesis replacing *gal4* with the *barhl1a* coding sequence. Transformation through electroporation of the *pRedET* plasmid was performed as described^59^. For Tol2 transposon-mediated BAC transgenesis, the iTol2-amp cassette^59^ was amplified by PCR with the primer pair pTarBAC_HA1_iTol2_fw (5′-gcgtaagcggggcacatttcattacctctttctccgcacccgacatag atCCCTGCTCGAGCCGGGCCCAAGTG-3′) and pTarBAC_HA1_iTol2_rev (5′-gcggggcatgactattggcgcgccggatcgatccttaattaagtctactaATTATGATCCTCTAGATCAGCTC-3′), where the lower and upper cases indicate the pTarBAC2.1 sequences for homologous recombination and the iTol2-amp annealing sequences, respectively. Subsequently, the amplified iTol2-amp cassette was introduced into the backbone (*pTarBAC2.1*) of the Barhl1a-BAC. 500 ng of the PCR product (1 mL) were used for electroporation. 5′-caaaaccagtgtcataaaggacaaatgcacatttgatattgatttgactcGCCACCATGAAGCTACTGTCTTCTATCGAAC-3′ and 5′-ctgtgagaaagtatagactcgatcccaaagctcgagccgtttgatacctcCCGCGTGTAGGCTGGAGCTGCTTC-3′ primers were used to amplify and insert the gal4FF cassette into the BAC^60^. The lower and upper cases indicate the CH211-215C18 sequences for homologous recombination and the pGal4FF-FRT-Kan-FRT annealing sequences, respectively. 500 ng of the PCR product (1 mL) were used for electroporation in Barhl1a-iTol2-amp-BAC -containing cells. The BAC DNA preparation was performed using the HiPure Midiprep kit (Invitrogen), with modifications for BAC DNA isolation as described by the manufacturer. Tol2 transposase mRNA was prepared by *in vitro* transcription from XbaI-linearized pDB600^61^ using the T3 mMessage mMachine kit (Ambion). RNA was purified using the RNeasy purification kit (Qiagen), diluted to a final concentration of 100 ng/μl for injection. At least 20 injected fish were backcrossed to wild type. Germline transmission was observed in the offspring from two of such crossings; which displayed the distribution pattern of GFP as expected from *barhl1a* expression in the posterior thalamus and zona limitans intratalamica (ZLI) (Supplementary Fig. 1A). Only one of the two F1 generations was used to carry out all of the following analyses.

The zebrafish *gfap* promoter^50^ was derived from the Addgene plasmid: *pEGFP-gfap*(Intron1/5′/Exon1-zebrafish) (Addgene, #39761) and *lssmKate2*^62^ was derived from the Addgene plasmid: pLSSmKate2-N1 (Addgene, #31867). In a first step the *gfap* promoter driving GFP was subcloned into a vector containing IsceI sites using NotI and XhoI restriction enzymes (NEB #R3189 and #R0146). To insert *lssmKate2* SalI and NotI restriction enzymes (NEB #R3138S and #R3189) were used, resulting in the following plasmid: *IsceI::gfap(Intron1/*5′*/Econ1-zebrafish)::LssmKate2 polyA*.

### Immunohistochemistry

The primary antibodies used in this study and their dilutions were the following: chicken anti-GFP antibody (Life Technologies, A10262, 1:500), rabbit anti-DsRed (Clontech, 632496, 1:200), mouse anti-Tubulin (Sigma T5168, 1:100), mouse anti-HUC (Molecular probes, A-21271, 1:100) rabbit anti-pH3 (Millipore, 06-570; 1:500), mouse anti-Zn5 (ZIRC Zn-5; 1:200), monoclonal zebrafish anti-Cxcr4b (1:100^63^. Secondary antibodies were goat or donkey anti-mouse, anti- rabbit or anti-goat IgG conjugated to Alexa Fluor 488, 546, 594 or 647 fluorophores (1:500 for whole-mount to 1:2000 dilutions for cryosections; Invitrogen).

Whole-mount immunohistochemistry experiments were carried out as follows: embryos were fixed 1 to 2 hours in 4% paraformaldehyde (4% PFA). The fixative was then washed out using 1X PBS-Tween solution. Permeabilization of the embryos was done on ice using 0.25% Trypsin-EDTA (with phenol red; Gibco, Life Technologies) solution. The duration of trypsin treatment was dependent on the stage of the embryos: 28–30 hpf: 3 min; 31–33 hpf: 4 min; 34–35 hpf: 6 min; 40 hpf: 7–8 min; 45 hpf: 9–10 min; 48–50 hpf: 11–12 min; 60 hpf: 20 min. Embryos were then washed several times using 0.1 M PBS-Tween solution (1X PBS-Tween solution), blocked using 10% blocking solution (10% heat-inactivated goat serum, 1% bovine serum albumin, 0.2% Triton X-100 in PBS) and incubated overnight at 4 degrees in 1% blocking solution (10% blocking solution in 1X PBS-Tween) in which primary antibodies were diluted. Primary antibodies were then washed out using 1X PBS-Tween and embryos were then incubated overnight at 4 degrees in 1% blocking solution (1% heat-inactivated goat serum, 1% bovine serum albumin, 0.2% Triton X-100 in PBS) in which secondary antibodies and 4′,6-diamidino-2-phenylindole (DAPI) were diluted. Prior to imaging, embryos were washed in 1X PBS-Tween and stored at 4 degrees.

For the immunohistochemistry on sections and RGC counting, 50 hpf *Tg*(*barhl1a:GFP;atoh7:gap43-RFP*) embryos were fixed in 4% paraformaldehyde (PFA) in 0.1 M phosphate saline buffer (PBS) overnight at 4 °C, rinsed and cryoprotected in 30% sucrose (w/v) overnight at 4 °C. Embryos were then mounted vertically (head down) in freezing medium (Jung Tissue Freezing Medium, Leica Microsystems), frozen in liquid nitrogen and cryosectioned immediately with Leica CM3050 S cryostat. The thickness of sections was 14 μm. Sections were collected on adhesion microscope slides (SuperFrost Plus, Menzel-Gläser) and left to dry overnight at 4 °C. For immunohistochemistry, microscope slides with cryosections were washed 3 times (each wash 15 min) in PTW, then covered with 10% goat blocking medium and incubated at room temperature for 1 h. The cryosections were then incubated overnight in primary antibodies: anti-chicken GFP (Life Technologies, A10262) and anti-mouse Zn5 (Zebrafish International Resource Center (ZIRC)) both diluted 1:500 in 1% goat blocking medium. Microscope slides were then washed again 3 times (each wash 15 min) in PTW and secondary antibodies were added: anti-chicken conjugated to Alexa Fluor 488 (from donkey; Jackson ImmunoResearch Laboratories, Inc., 703-545-155) and anti-mouse conjugated to Alexa Fluor 546 (from goat; Invitrogen, A-11030) both diluted 1:500 in 1% goat blocking medium. Cryosections were incubated with the secondary antibody mix for 1.5–2 h at 37 °C in the dark and then washed again in PTW (3 times 15 min) at room temperature. They were then incubated in DAPI (10 μg/ml in PTW) for 10–15 min and washed in PTW (3 times 15 min). Microscope slides were then dried from the back and edges with a paper tissue, 120 μL 60% glycerol was added and cryosections were covered with coverslips (24 × 60 mm, Carl Roth). Coverslips were sealed with nail polish and microscope slides were stored at 4 °C in the dark until imaging.

### Double fluorescent *in situ* hybridization

For *barhl1a/GFP* double fluorescent whole mount *in situ* hybridization (FISH), standard digoxigenin- and fluorescein-labeled riboprobes were combined with Tyramide Signal Amplification, as described by^10^. *Barhl1a* riboprobe was synthesized as previously reported^10^ and *gfp* riboprobe was synthesized from a linearized *pCS2:GFP* plasmid with NotI (Fermentas or New England Biolabs) and transcribed with Sp6 (mMessage mMachine Sp6, Ambion). Incubation with the *barhl1a* probe was for 40 min, incubation with the *gfp* probe was for 30 min. Embryos were then kept in the dark for all following steps. For detection and staining of the antisense probes, embryos were washed 5 times 10 min with TNT (0.1 M Tris pH7.5, 0.15 M NaCl, 0.1% Tween-20), incubated with 1% H_2_O_2_ in TNT for 20 min and washed again 5 times 10 min. Embryos were blocked in TNB (2% DIG Block in TNT) for 1 h at room temperature and afterwards incubated with Anti-Digoxigenin-POD Fab fragments diluted 1:100 in TNB. For signal detection, Fluorescein (FITC), Cyanine 3 (Cy3) or Cyanine 5 (Cy5) Fluorophore Tyramide by PerkinElmer was used. Embryos were then incubated in 1 × 4′,6-Diamidin-2-phenylindol (DAPI) in TNT overnight at 4 °C and washed several times in TNT the next day. The stained embryos were then used for imaging or kept in the dark at 4 °C until imaging.

For *barhl1a/lmx1b1* double fluorescent *in situ* whole mount hybridization (FISH), antisense RNA probes for *barhl1a* and *lmx1b1* were produced from full-length cDNA clones generated by^64^. Antisense probes were generated as described in^64^ except that DNP-11-UTP (Perkin Elmer) was used for the production of *barhl1a* RNA probe. Fluorescent double whole mount FISH, RNA hybridization were performed on dechorionated 20 hpf embryos with the tyramide amplification kit (TSA Plus Cyanine 3 System, Perkin Elmer, Boston, MA). Briefly, manually dechorionated 20 hpf embryos were fixed in 4% (w/v) PAF for 3 h at 4 °C and conserved until use in 100% (v/v) Methanol at −20 °C. Embryos were rehydrated through MetOH/PBS gradient series and washed 3 times in 0.1% (v/v) Tween 20 in PBS buffer (PTW). Endogenous peroxidase were inactivated in 2% (v/v) H_2_O_2_, the embryos incubated 2 min with proteinase K (10 μg/ml at room temperature (20 °C), then post-fixed 15 min in 4% PFA for 30 minutes at room temperature and washed 5 times 5 min in PBS-Tween. Embryos were then pre-hybridized 3 hours before overnight incubation at 65 °C in a hybridization buffer (pH 6) containing the antisense-labeled probes. On the second day, embryos were washed in post-hybridization buffers, incubated 1 h in the blocking buffer and then incubated overnight with an anti-DNP-POD antibody (1:1000, Perkin Elmer). The next day embryos were washed in PBS-Tween, and stained with tyramide Cy3 solution (1:100) in 0.002% (v/v) H_2_O_2_ in PBS-Tween, peroxidase were inactivated in 2% (v/v) H_2_O_2_ for 1 hour and washed in PBS-Tween. The same day, embryos were incubated overnight with an anti-digoxigenin-poly-POD antibody (1:1000, Roche), then washed in PTW and stained with tyramide Cy5 solution (1:100) in 0.002% (v/v) H_2_O_2_ in PBS-Tween.

Of note, FISH for *GFP* mRNA probe can give unspecific signals detected in the peripheral retina and lens^20^.

### Imaging and quantification

For either imaging of fixed embryos or live imaging, embryos were embedded in 1% low-melting agarose (w/v, diluted in H_2_O; Biozym) in 35 mm Glass-bottom Microwell dishes (P35G-1.5-10-C, MatTek). They were oriented with truncated Microloader tips (Eppendorf) frontally (head touching the dish and body axis tilted about 45 °C) or laterally. After solidification, agarose was covered with a drop of water to avoid dehydration during imaging. Confocal microscopy was carried out with Leica SpE or Leica Sp5 confocal laser scanning microscope using a Leica 20× or 60 × 1.2 NA water-immersion objective and Leica Application Suite (LAS) software. In most cases, confocal images were taken sequentially from the whole head/retina, with a distance of 1 to 5 μm in z-direction. Optical stack of 40–60 μm for time-lapse and a maximum of 100μm for fixed embryos were imaged. Live imaging was performed as previously described^21–23^. Images were taken every 5 or 10 minutes for 24–42 h and the motorized XY stage was used to image multiple embryos. For all imaging experiments, laser power was minimized as much as possible to avoid bleaching and photo-toxicity. Sequential image acquisition was also performed using individual descanned Leica PMT detectors. All image processing and 3D reconstructions were done using either Volocity 6.0.1 (PerkinElmer) or Fiji (https://imagej.net/ImageJ). All data presented in this study derive from observations made in at least 10 embryos/experiment in the case of fixed samples and on at least 3 embryos/experiment in the case of live imaging.

In order to quantify the number of Barhl1a:GFP cells in the GCL, 14 µm cryosections of 50 hpf (*barhl1a:GFP;atoh7:gap43-RFP*) double transgenic embryos were immunostained against GFP and Zn5 (also known as Alcama/DM-GRASP/Neurolin/Zn8), a cell adhesion molecule that is transiently found on the entire surface of differentiated RGCs^30^. Barhl1a:GFP positive cell nuclei were counterstained with DAPI. Sections were imaged with a Leica DM5000B compound microscope (20x objective). Three most central sections of each retina (n = 7 retinae from 6 embryos) were imaged. Both GFP positive and GFP negative nuclei were counted manually in Volocity Analysis version 5.3 (Improvision). To exclude cells in the periphery, where the CMZ continues to give rise to all retinal cell classes, only cells within the central quadrant of the retina were counted as previously described^20^. For every retina, the percentage of GFP-positive cells was calculated as a mean of the proportions observed in each individual section.

## Supplementary information

Supplementary Information.

Supplementary Information2.

Supplementary Information3.

Supplementary Information4.

Supplementary Information5.

Supplementary Information6.

Supplementary Information7.

Supplementary Information8.

Supplementary Information9.
