# Supplementary material for: Expression of a Barhl1a reporter in subsets of retinal ganglion cells and commissural neurons of the developing zebrafish brain
Source: Sci Rep. 2020 Jun 1;10:8814. doi: 10.1038/s41598-020-65435-w (PMC7264323; doi:10.1038/s41598-020-65435-w)
Supplement: Supplementary file 1 — Supplementary Information. [file 41598_2020_65435_MOESM1_ESM.docx]

Table 1. Quantification of Barhl1a:GFP-positive cells in the ganglion cell layer (GCL) of *Tg*(*barhl1a:GFP*) cryosectioned retinae at 50 hpf (7 retinae, 19 sections).

|  | Cryosection (central retina) | Nb. GFP cells in the GCL | Total number of cells in the GCL | GFP/Total in the GCL (%) |
| --- | --- | --- | --- | --- |
| Retina 1 | 1 | 48 | 99 | 48 |
|  | 2 | 31 | 76 | 41 |
|  | 3 | 51 | 89 | 57 |
| Retina 2 | 1 | 44 | 77 | 57 |
|  | 2 | 43 | 90 | 48 |
|  | 3 | 48 | 99 | 48 |
| Retina 3 | 1 | 36 | 67 | 54 |
|  | 2 | 49 | 89 | 55 |
|  | 3 | 38 | 79 | 48 |
| Retina 4 | 1 | 46 | 88 | 52 |
|  | 2 | 45 | 73 | 62 |
|  | 3 | 42 | 69 | 61 |
| Retina 5 | 1 | 44 | 82 | 54 |
|  | 2 | 37 | 60 | 62 |
|  | 3 | 31 | 51 | 61 |
| Retina 6 | 1 | 32 | 64 | 50 |
|  | 2 | 38 | 69 | 55 |
|  | 3 | 43 | 70 | 61 |
| Retina 7 | 1 | 31 | 61 | 51 |
| Total | 19 | 746 | 1391 | **54%** |
