# Supplementary figures and images for: Expression of a Barhl1a reporter in subsets of retinal ganglion cells and commissural neurons of the developing zebrafish brain
Source: Sci Rep. 2020 Jun 1;10:8814. doi: 10.1038/s41598-020-65435-w (PMC7264323; doi:10.1038/s41598-020-65435-w)

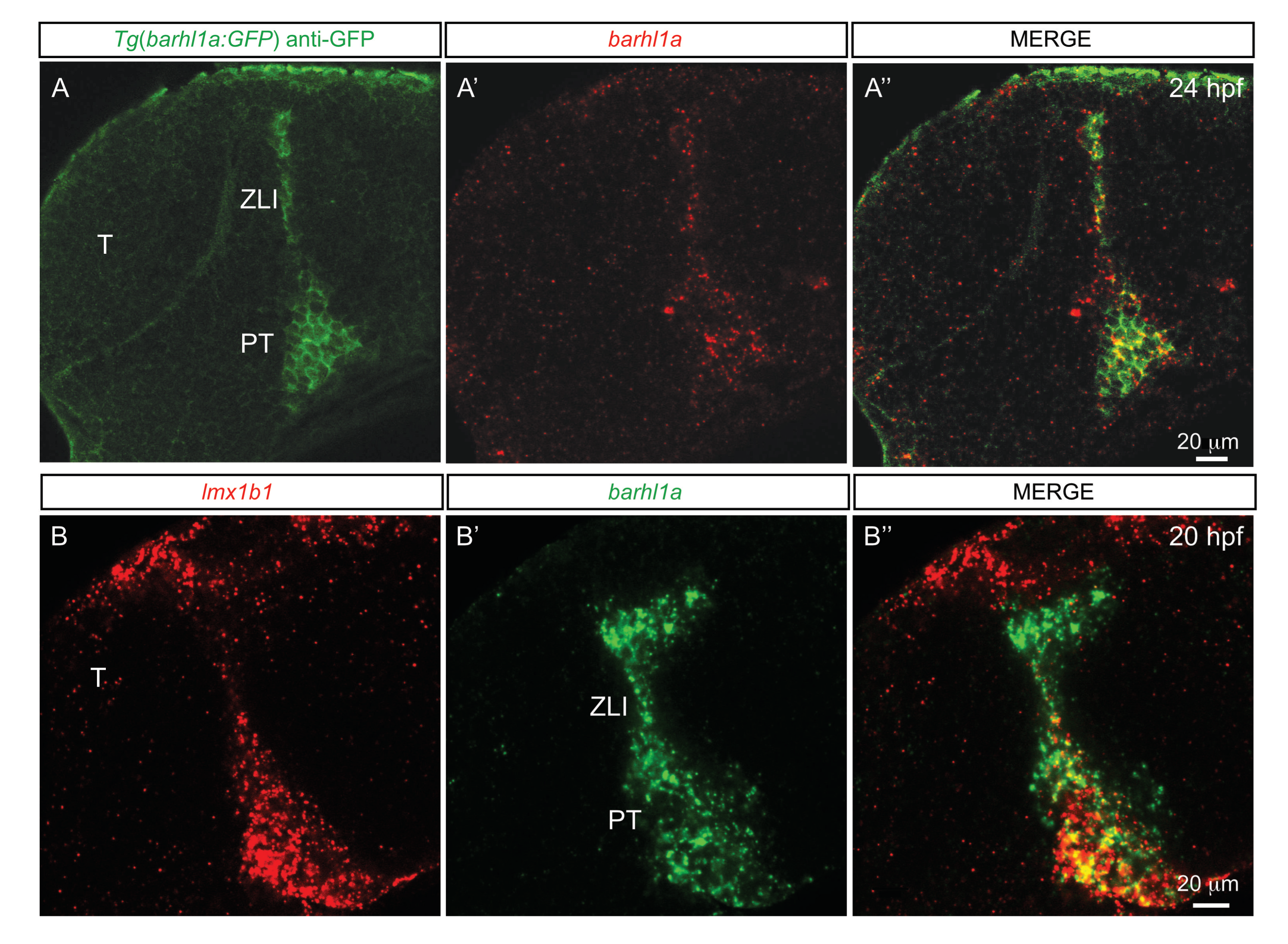

Supplement: Supplementary file 2 — Supplementary Information2. [file 41598_2020_65435_MOESM2_ESM.tiff]
